# Inflammatory Responses to *Salmonella* Infections Are Serotype-Specific

**DOI:** 10.1155/2013/168179

**Published:** 2013-09-16

**Authors:** Zhanna Ktsoyan, Karine Ghazaryan, Gayane Manukyan, Anush Martirosyan, Armine Mnatsakanyan, Karine Arakelova, Zaruhi Gevorgyan, Anahit Sedrakyan, Ara Asoyan, Anna Boyajyan, Rustam Aminov

**Affiliations:** ^1^Institute of Molecular Biology National Academy of Sciences of Republic of Armenia, Hasratyan 7, 0014 Yerevan, Armenia; ^2^“Nork” Clinical Hospital of Infectious Diseases, Ministry of Health of Republic of Armenia, Armenakyan 153, 0047 Yerevan, Armenia; ^3^Faculty of Medical Sciences, University of the West Indies, Mona, Kingston 7, Jamaica

## Abstract

The main purpose of this study was to investigate the profile of inflammatory response in patients with acute salmonellosis caused by two serotypes of *Salmonella enterica*, *S.* Enteritidis and *S.* Typhimurium, as well as in convalescent patients with previous acute disease caused by *S.* Enteritidis. Patients with acute disease showed significantly elevated levels of IL-1*β*, IL-17, IL-10, and calprotectin compared to healthy control subjects. In convalescent patients, these markers were also significantly elevated, with the exception of IL-1*β*. Multivariate statistical analyses with the use of these variables produced models with a good predictive accuracy resulting in excellent separation of the diseased and healthy cohorts studied. Overall, the results suggest that the profile of inflammatory response in this disease is determined, to a significant degree, by the serotype of *Salmonella*, and the profile of certain cytokines and calprotectin remains abnormal for a number of months following the acute disease stage.

## 1. Introduction

Infections by various *Salmonella* species remain one of the leading causes of gastrointestinal disorders in the world resulting in significant morbidity and some mortality [1]. Infections by the two most common serotypes of *Salmonella enterica*, *S.* Enteritidis and *S.* Typhimurium, are the most frequent causes of acute gastroenteritis in humans worldwide. Although the members of the *Salmonella *genus are genetically close, they show wide variations in host-specificity, virulence, and disease manifestations [2]. Clinical picture of the disease depends on the host, the serotype of *Salmonella*, and the specificity of the interaction of certain serotypes with the host, but not all details of these complex interactions are fully understood.

Under the normal physiological conditions of a healthy host, the gut defense system maintains several protective barriers to keep the bacteria at bay [3]. This network of defense mechanisms at mucosal surfaces is regulated by a number of regulatory signals, including cytokines, to maintain the controlled inflammation state under the normal circumstances. In the case of invasive pathogens such as *Salmonella*, the cytokines play an important protective role orchestrating a number of events leading to the local inflammatory response followed by its downregulation once the body is cleared from a pathogen [4].

The inflammatory response also includes the synthesis of antimicrobial peptides, some of which may possess a secondary role as regulatory molecules [5]. When the immune system detects the presence of a pathogen and the inflammatory cascade is initiated, the infected area is flooded by neutrophils, with more than 50% of total proteins in the cytosol of neutrophil granulocytes being calprotectin. The main mechanism of antimicrobial activity of calprotectin is thought to be mediated through the sequestration of essential micronutrient metals such as zinc and manganese, thereby limiting their availability to bacteria [6], in a process termed “nutritional immunity” [7]. In the case of gastroenteritis caused by *S.* Typhimurium infection, the luminal content displays the elevated concentrations of calprotectin although the calprotectin-mediated zinc chelation can be circumvented by the pathogen by expressing a high-affinity zinc transporter, ZnuABC [8]. In addition to the metal chelation, this complex displays other functions such as a leukocyte chemoattractant, an oxidant scavenger, and a chemokine-like factor [9]. This multifunctional complex is also involved in the induction of apoptotic processes in the host due to zinc depletion [10]. Also, by scavenging zinc, calprotectin can inhibit many important metalloproteinases that are involved in normal physiological processes as well as in inflammatory tissue damage [11]. Thus, this protein serves as a good marker for a number of diseases involving inflammation component such as rheumatoid arthritis, inflammatory bowel disease, cancer, and various infections.

There is a paucity of information regarding the regulation of inflammation by cytokines and antimicrobial peptides during salmonellosis. The main purpose of this study was to investigate the profile of inflammatory response in relation to the serotype of the pathogen and the stage of salmonellosis. With the use of enzyme-linked immunosorbent assay (ELISA), we examined the levels of IL-1*β*, IL-17, IL-10, and calprotectin in the blood serum of patients with acute salmonellosis caused by *S.* Typhimurium and *S.* Enteritidis as well as in the convalescent patients after the acute disease caused by *S.* Enteritidis. The control group comprised healthy volunteers. 

## 2. Materials and Methods

The study groups included patients with salmonellosis admitted to the infectious disease hospital (Nork) in Yerevan, RA. A total of 64 patients with salmonellosis caused by *S.* Typhimurium (*n* = 30, acute disease) and *S.* Enteritidis (*n* = 34, acute disease) were enrolled in this study. The convalescent state (*n* = 10) was investigated in the subgroup of *S.* Enteritidis-infected patients, who were followed up after the acute disease for one to seven months. The gender and age distribution in the *S.* Typhimurium group was as follows: 12 males and 18 females, median age—2.4 years old (interquartile range (IQR) 1.5–3.9, range 0.3–25.0). In the *S.* Enteritidis group it was follows: 17 males and 17 females, median age—5.40 years old (IQR 2.3–14.8, range 1.1–27.0). Diagnosis was based on clinical presentations and laboratory analyses. Clinical presentations consistent with gastroenteritis were diarrhea, fever, nausea, vomiting, and abdominal cramps. Anamnesis of food consumed, water sources, social gatherings, anybody else with a similar illness, and any recent travel was also recorded. The convalescent group included the former patients who experienced the acute disease caused by *S.* Enteritidis from one to six months ago and who attended the follow-up visit to the hospital. The median time point of the follow-up visit after the discharge was 5 months (IQR 4.0–5.8, range 3.0–6.0). At the time of the visit, blood and fecal samples were collected. These samples were negative for *Salmonella*. The gender and age distribution in this group was as follows: 4 males and 6 females, median age—3.2 years old (IQR 2.0–4.8, range—1.6–17.0). The control group included 25 healthy volunteers with no discernible disease and not taking any medication including antibiotics. The gender and age distribution in this group was as follows: 11 males and 14 females, median age—7.0 years old (IQR 4.5–23.0, range 3.0–39.0).

For this investigation, only the patients who were not taking any type of medication including antibiotics before the hospital admission were chosen. Blood and fecal samples have been taken on the first or the second day of hospital admission. At the time of discharge from the hospital, no *Salmonella* strains have been detected in the fecal samples or blood of any of the patients. For detoxification and rehydration, all patients were subjected to the standard infusion therapy. Based on the severity of the disease, 86% were placed on antibiotic therapy: (i) 38% received intravenous or intramuscular ceftriaxone (50 mg/kg/day); (ii) 42% received peroral ciprofloxacin (0.5–1.0 g/day); and (iii) the rest were on the combination of one of the two drugs and trimethoprim/sulfamethoxazole. All study subjects, or their parent or guardian if a child, gave their written consent to give fecal and blood samples for the study. The study protocol was approved by the Ethics Committee of the Institute of Molecular Biology NAS RA (IORG number 0003427, Assurance number FWA00015042, and IRB number 00004079).

Biochemical tests for the identification of *Salmonella* were fermentation of glucose, negative urease reaction, lysine decarboxylase, negative indole test, H_2_S production, and fermentation of galactitol (dulcitol). Serotypes of *Salmonella* were determined using the standard Kauffman-White scheme with the use of commercially available polyvalent antisera for flagellar (H) and for lipopolysaccharide (O) antigens. The isolates were tested for susceptibility to eight classes of antibiotics by disc diffusion method according to the guidelines of the Clinical and Laboratory Standards Institute (CLSI). Commercially available discs were used (Micromaster Laboratories Pvt. Ltd., India), and the antibiotics used were streptomycin, gentamicin, amoxicillin/clavulanic acid, ceftriaxone, trimethoprim/sulfamethoxazole, sulfisoxazole, ampicillin, chloramphenicol, ciprofloxacin, and tetracycline.

Concentrations of IL-1*β*, IL-10, and IL-17 in the serum samples were determined with the ELISA plates (Pierce Biotechnology) according to the manufacturer's protocols. Concentration of calprotectin in the sera was evaluated using the ELISA (serum) kit by DRG Diagnostics (Germany), according to the manufacturer's instructions. The plates were read on a Stat Fax 303 Reader. Calibration curves for determining the concentrations of interleukins and calprotectin in experimental samples were obtained using the standards included in the kits. Absorbency readings were performed on a Stat Fax 303 Reader (Awareness Technology, Inc., USA). Detection limits for the assays were IL-1*β*—1 pg/mL, IL-10—3 pg/mL, and IL-17—<4 pg/mL. 

Statistical analyses were performed using the software package “GraphPad QuickCalcs: *t* test calculator” (GraphPad Software Inc., USA). Discriminant function analysis (DA) was performed using the SPSS package (SPSS Inc., Chicago, IL, USA). Subjects in each cohort were taken as dependent variables of DA, and the concentrations of interleukins and calprotectin were taken as predictors. *P* values below 0.05 were considered statistically significant.

## 3. Results

With the use of ELISA, we investigated the levels of IL-1*β*, IL-17, IL-10, and calprotectin among the patients with salmonellosis caused by *Salmonella enterica* serovar Typhimurium (acute disease) and *S. enterica* serovar Enteritidis (acute disease) as well as in the convalescent group and in healthy volunteers. We found a statistically significant increase of almost all cytokines and calprotectin in patients compared to healthy control subjects (Table 1). 

Comparative analysis showed a statistically significant difference in the concentration of systemic IL-1*β*, IL-17, and calprotectin in acute disease caused by two different serotypes of *Salmonella* (Table 1). In particular, the concentration of IL-1*β* was higher in patients infected with *S.* Typhimurium, while that of IL-17 was higher in patients infected with *S.* Enteritidis. Interestingly, the systemic calprotectin levels were also higher in patients infected with *S.* Enteritidis compared to patients infected with *S.* Typhimurium. The level of systemic IL-10 was consistently higher in acute disease and also in the convalescent state compared to healthy controls (Table 1).

**Figure 1:**
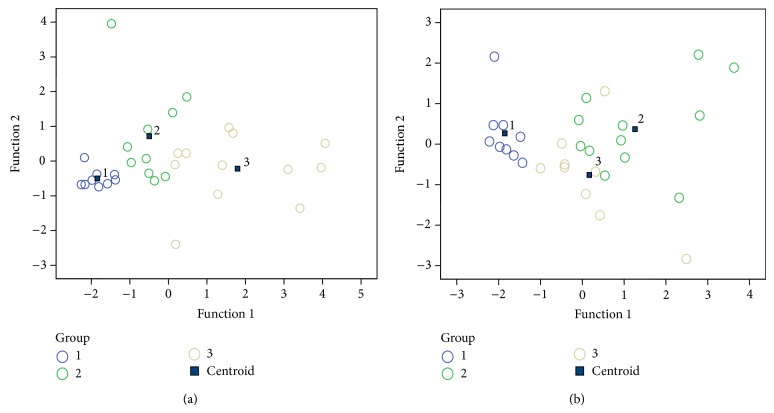
Scatterplot of DA model based on systemic concentration of interleukins and calprotectin in salmonellosis patients and controls. The number of variables in the model—4 (IL-17, IL-1*β*, IL-10, and calprotectin) and grouping consists of three groups. Roots 1 and 2—discriminant functions 1 and 2 (1st and 2nd canonical roots). Group centroid positions are numbered: (a) 1—healthy control subjects, 2—patients with acute *S.* Typhimurium infection, and 3—patients with acute *S.* Enteritidis infection; (b) 1—healthy control subjects, 2—patients with acute *S.* Enteritidis infection, and 3—patients in the convalescent state following the previous acute *S.* Enteritidis infection.

The raw data were further subjected to multivariate statistical analysis to determine the distribution of patterns from the complex datasets obtained from each group studied (Figure 1, Table 2). The results demonstrated that the DA model has a good predictive accuracy resulting in excellent separation of the diseased and healthy cohorts studied. In particular, 90.3% of the original group cases were correctly classified based on the four variables used (Table 2(A)). Predicted classification of the disease stage was also confident at 86.7% (Table 2(B)). Thus, the results of discriminant function analysis suggest that the profile of inflammatory response in salmonellosis is determined, to a significant degree, by the serotype of the causative agent and by the disease stage.

Antibiotic susceptibility tests indicated that 17.6% of *S.* Enteritidis isolates and 3.3% of *S. *Typhimurium isolates were sensitive to all antibiotics tested. The level of sensitivity of *S.* Enteritidis isolates towards the antibiotics used in the therapy was ciprofloxacin—76.5% (MIC ≤ 0.06 mg/L), ceftriaxone—64.7% (MIC ≤ 1.0 mg/L) and trimethoprim/sulfamethoxazole—79.3% (MIC ≤ 2.0/38.0 mg/L). The corresponding sensitivity values for *S.* Typhimurium isolates were: ciprofloxacin 93.3% (MIC ≤ 0.06 mg/L), ceftriaxone 33.3% (MIC ≤ 1.0 mg/L), and trimethoprim/sulfamethoxazole 36.7% (MIC ≤ 2.0/38.0 mg/L).

## 4. Discussion

In this work, the concentration of systemic IL-1*β*, IL-10, IL-17, and calprotectin was measured in patients with acute disease caused by *S.* Enteritidis or *S.* Typhimurium infection as well as in convalescent patients with the previous acute *S.* Enteritidis infection compared to the healthy state. The results suggest that the profile of inflammatory response is largely driven by the serotype of the pathogen, and the profile of certain cytokines and calprotectin remains abnormal for a number of months following the acute disease stage.

It is not entirely unexpected that the infection by both serotypes of *Salmonella* and the following acute disease is associated with the sharp increase in systemic concentration of IL-17 (Table 1). IL-17 is a central cytokine implicated in inflammation and antimicrobial defense [12]. In the murine models of *S.* Enteritidis infection, the onset of enterocolitis is associated with a dramatic increase in the expression of IL-17 [4, 13]. Previous studies revealed an important role played by the Th17-mediated mucosal immunity in the early control of infections caused by a variety of pathogens [12, 14, 15]. The responses elicited by Th17 cytokines contribute to the mucosal barrier by preventing the attaching and effacing of bacterial pathogens, containing an infection, and preventing dissemination of pathogens to systemic sites.

Th17-produced interleukins IL-17 and IL-22 also induce a number of antimicrobial peptides including calprotectin [5, 16]. In our survey, the concentration of systemic calprotectin in acute disease was following the IL-17 trend and was consistently high, exceeding the normal values by four- to sevenfold (Table 1). As mentioned before, calprotectin is a multifunctional complex and its antimicrobial activity is thought to be realized through the nutrition immunity mechanism [6]. Paradoxically, however, zinc sequestration by calprotectin enhances *Salmonella* growth in the inflamed gut because of the expression of a high-affinity zinc transporter by the bacterium that allows alleviating the zinc deficiency [8]. Zinc deficiency, though, may still have a protective effect enforcing the infected host cells in the local microenvironment into the apoptotic route, thus destroying the pathogen as well. Also, these peptides may be both mediators and end-effectors in this cytokine-regulated commitment to inflammation and proliferation, as indicated in the paradigm of the crosstalk between innate immune cells and Th17 cells, and express chemokine-like effects [9].

The level of induction of IL-17 during acute disease is serotype-dependent: infection by *S.* Enteritidis results in twice as much of systemic IL-17 than in the case of *S.* Typhimurium (Table 1). Taking into consideration the IL-17-mediated induction of antimicrobial peptides, the level of calprotectin in patients infected by *S.* Enteritidis is also proportionally higher than that in the latter cohort. Despite the strong induction of IL-17A and IL-17F in the mucosa of *S.* Typhimurium-infected mice, the IL-17RA signaling seems to be dispensable for eliciting the acute disease [17]. This serotype, therefore, may induce other than IL-17 cytokines in the network that are more important in mediating inflammatory response. 

In contrast, the opposite trend in acute disease is observed for the concentration of systemic IL-1*β*: in patients infected with *S.* Typhimurium it is 2.3-fold (*P* = 0.01585) higher than that in the group of patients infected with *S.* Enteritidis. This interleukin is implicated in multiple immune reactions including the recruitment of inflammatory cells such as monocytes, macrophages, and neutrophils to the sites of infection [18]. It also activates the release of other proinflammatory cytokines and induces a Th17 bias in the cellular adaptive responses [19]. The stages leading to the induction, maturation, and secretion of IL-1*β* are complex and include two independent signals: (i) induction of pro-IL-1*β* by microbial stimuli via TLR or NOD2 stimulation and NF-*κ*B activation [20]; (ii) sensing the microbial and endogenous danger signals by the NOD-like receptor components of the inflammasomes and activation of caspase-1, which then cleaves pro-IL-1*β* into the biologically active form [21]. 

A number of Gram-negative bacteria, including *S.* Typhimurium, induce the synthesis of pro-IL-1*β* through the initial signaling involving the extracellular TLR4 and TLR5 receptors. The second stage, the induction of caspase-1 to process pro-IL-1*β* into the mature form by *S.* Typhimurium, requires the presence of flagellin and the rod protein in the cytosol, which are recognized by the NLRC4 inflammasome; the flagellin in this case is recognized by some other intracellular receptor(s) other than the extracellular TLR5 receptor [22–24]. Signaling through the NLRC3 inflammasome by *S.* Typhimurium also plays a role in the activation of caspase-1, although the molecular mechanisms of this induction are unknown [25]. Much less is known about the induction of IL-1*β* by *S.* Enteritidis. Perhaps, the similar IL-1*β* induction pathway involving flagellin as a signaling molecule exists in this serotype as well because the inactivation of the corresponding gene strongly reduces its pathogenic properties such as invasiveness [26]. A different way of IL-1*β* induction may be also realized by this serotype since the disruption of a gene that is specific for this serotype and encodes a protein with homology to the mammalian Toll/interleukin-1 receptors leads to the decreased IL-1*β* secretion [27]. The activation of IL-1*β* during *Salmonella* infection is a complex process employing several different pathways [28], and it is difficult to discern the contribution of each process to the net systemic concentration of IL-1*β*. But overall, *S.* Typhimurium is a more potent inducer of IL-1*β* than *S.* Enteritidis.

The concentration of systemic IL-10 in patients with salmonellosis is significantly higher compared to control subjects (Table 1). However, there is no statistically significant difference among the diseased cohorts. In general, this anti-inflammatory cytokine is considered as an essential immunoregulator in the intestinal tract [29]. Its main function is to counterbalance an overly zealous proinflammatory response to protect the host from its harmful side effects [30]. On the other hand, this activity may result in the persistence of bacteria and viruses due to the interference with the innate and adaptive protective immunity [31]. In the case of *S.* Typhimurium infection, IL-10 not only suppresses the bactericidal response of macrophages against the pathogen, but also ultimately causes infected macrophages to function as hosts for its replication [32]. Another consequence is that infections causing the expansion of interleukin-10-producing regulatory cells may have protective effects against allergic diseases [31]. 

Convalescent state in our study is defined as the recovery stage following acute *Salmonella* infection. Need to note here that these patients were *Salmonella*-free at the time of discharge from the hospital after the initial acute episode of the disease as well as during the follow-up visit. The convalescent state following acute *S.* Enteritidis infection is characterized by the pattern of inflammation that is different from the acute stage of the disease (Table 1, Figure 1). In our patients, we were unable to discern any particular factor, except the serotype, that could be considered as a main factor shaping the particular inflammatory profile of the convalescent state. Need to note here that the convalescent state is not associated with the asymptomatic carriage, all patients were *Salmonella*-free. Most likely, this inflammatory profile is a consequence of the perturbed host responses during the acute disease that persists for long time after the clearance of infection.

Multivariate statistical analyses revealed the leading role of two factors, the serotype of *Salmonella* and the disease stage, in inducing a specific set of cytokines and an antimicrobial protein (Figure 1). Differential genomic context of the serotypes may explain the differential induction of inflammatory responses. Infection of mice with *S.* Typhimurium and *S.* Enteritidis involves a subset of genes common for both serotypes, the latter, however, employs a number of additional genes that are absent in the genome of the former serotype such as a type I restriction/modification system, a fimbrial operon, a putative pathogenicity island, and a type VI secretion system remnant, encoding a hypothetical protein containing a lysine motif [33]. Thus, the differential gene content/expression may explain the differential induction of inflammatory responses by these two serotypes.

In conclusion, our results suggest that the induction of the cytokine network and an antimicrobial protein is serotype-specific and also depends on the disease stage. This conclusion is in agreement with a previous suggestion that the outcome of salmonellosis is largely dependent on the serotype [34]. Although *Salmonella* serotypes are genetically very close, there is a substantial variation among them in pathogenic potentials as well as in host responses to the infection. Further investigations are necessary to clarify the role of genes differently represented/expressed in the genomes of various *Salmonella* serotypes during infection.

## Figures and Tables

**Table 1:** Concentration of systemic interleukins (pg/mL) and calprotectin (ng/mL).

Interleukins and calprotectin	Healthy control subjects (*n* = 25)	Patients		
		Acute disease		Convalescent
		*S*. Typhimurium (*n* = 30)	*S*. Enteritidis (*n* = 34)	*S*. Enteritidis (*n* = 10)
IL-17	3.6 ± 0.8^*^	10.4 ± 2.3^x^	22.8 ± 3.2^xy^	10.7 ± 2.2^y^
IL-1*β*	1.6 ± 0.5	6.7 ± 1.6^xz^	2.9 ± 0.3^xyz^	1.5 ± 0.3^y^
IL-10	3.8 ± 1.1^*^	15.2 ± 5.3	23.1 ± 3.0	26.0 ± 2.3
Calprotectin	6.9 ± 0.9^*^	27.5 ± 3.9^x^	51.2 ± 6.8^xy^	17.9 ± 3.4^y^

^*^Statistically significant difference compared to acute disease and convalescent group (*P* < 0.05).

^x^Difference between the *S*. Typhimurium and *S*. Enteritidis acute infections is statistically significant (*P* < 0.05).

^y^Difference between the acute disease and convalescent group is statistically significant (*P* < 0.05).

^z^Statistically significant difference compared to healthy control (*P* < 0.05).

**Table 2:** Predicted classification of patients and controls based on DA model.

Group		Predicted group membership			Total
		1	2	3	
(A) 90.3% of the original group cases were correctly classified; Wilks' Lambda = 0.218; *P* < 0.0005					
Count	1 Healthy controls	9	0	0	9
	2 Patients with acute *S*. Typhimurium infection	0	10	0	10
	3 Patients with acute *S*. Enteritidis infection	0	3	9	12
%	1 Healthy controls	100	0	0	100
	2 Patients with acute *S*. Typhimurium infection	0	100	0	100
	3 Patients with acute *S*. Enteritidis infection	0	25	75	100

(B) 86.7% of the original group cases were correctly classified; Wilks' Lambda = 0.274; *P* < 0.0005					
Count	1 Healthy controls	9	0	0	9
	2 Patients with acute *S*. Enteritidis infection	0	9	3	12
	3 Convalescent patients	0	1	8	9
%	1 Healthy controls	100	0	0	100
	2 Patients with acute *S*. Enteritidis infection	0	75	25	100
	3 Convalescent patients	0	11.1	88.9	100
